# An Amphiphysin-Like Domain in Fus2p Is Required for Rvs161p Interaction and Cortical Localization

**DOI:** 10.1534/g3.115.023960

**Published:** 2015-12-16

**Authors:** Richard A. Stein, Jean A. Smith, Mark D. Rose

**Affiliations:** Department of Molecular Biology, Princeton University, New Jersey 08544

**Keywords:** conjugation, karyogamy, BAR domain

## Abstract

Cell–cell fusion fulfils essential roles in fertilization, development and tissue repair. In the budding yeast, *Saccharomyces cerevisiae*, fusion between two haploid cells of opposite mating type generates the diploid zygote. Fus2p is a pheromone-induced protein that regulates cell wall removal during mating. Fus2p shuttles from the nucleus to localize at the shmoo tip, bound to Rvs161p, an amphiphysin. However, Rvs161p independently binds a second amphiphysin, Rvs167p, playing an essential role in endocytosis. To understand the basis of the Fus2p–Rvs161p interaction, we analyzed Fus2p structural domains. A previously described N-terminal domain (NTD) is necessary and sufficient to regulate nuclear/cytoplasmic trafficking of Fus2p. The Dbl homology domain (DBH) binds GTP-bound Cdc42p; binding is required for cell fusion, but not localization. We identified an approximately 200 amino acid region of Fus2p that is both necessary and sufficient for Rvs161p binding. The Rvs161p binding domain (RBD) contains three predicted alpha-helices; structural modeling suggests that the RBD adopts an amphiphysin-like structure. The RBD contains a 13-amino-acid region, conserved with Rvs161p and other amphiphysins, which is essential for binding. Mutations in the RBD, predicted to affect membrane binding, abolish cell fusion without affecting Rvs161p binding. We propose that Fus2p/Rvs161p form a novel heterodimeric amphiphysin required for cell fusion. Rvs161p binding is required but not sufficient for Fus2p localization. Mutations in the C-terminal domain (CTD) of Fus2p block localization, but not Rvs161p binding, causing a significant defect in cell fusion. We conclude that the Fus2p CTD mediates an additional, Rvs161p-independent interaction at the shmoo tip.

Cell fusion is a highly conserved eukaryotic process, with fundamental roles in fertilization, as well as development, disease pathogenesis, and tissue repair (Buckingham 2006; Linder 2007; Wassarman and Litscher 2008; Kim *et al.* 2015). Placental trophoblast formation and muscle development are two examples of processes containing post fertilization cell fusion events (Mi *et al.* 2000; Taylor 2002; Primakoff and Myles 2002). Understanding cell fusion could aid attempts to develop therapies targeting muscle degenerative disorders or other myopathies (Gibson *et al.* 1995).

Ultrastructural studies suggest that cell fusion events are morphologically similar in several distinct cell types. In cells surrounded by an extracellular matrix, fusion of the plasma membrane must be preceded by removal of the intervening material. In the budding yeast *Saccharomyces cerevisiae*, prior to plasma membrane fusion, cell wall thinning occurs at the site of contact between the two haploid fusion partners, coincident with the accumulation of electron-dense vesicles at the zone of cell fusion (ZCF) (Gammie *et al.* 1998). Similarly, in the *Drosophila* myoblast prefusion complex, electron-dense vesicles accumulate on the cytoplasmic faces of the apposed plasma membranes of the two cells (Doberstein *et al.* 1997; Estrada *et al.* 2007; Sens *et al.* 2010).

In *S. cerevisiae*, the mating pathway begins when two haploid partners of opposite mating types (*MAT***a** and *MAT*α) detect secreted mating pheromone from the other cell. Pheromone binding initiates a G-protein-coupled response, which activates a MAP kinase signal transduction pathway. This pathway results in multiple downstream responses including G1 cell cycle arrest, transcription of genes involved in mating, and polarization toward the mating partner (Dohlman and Slessareva 2006; Merlini *et al.* 2013). After cell-cycle arrest, the two partners extend mating projections, creating pear-shaped cells called shmoos, and contact each other to form a prezygote. The prezygotes degrade the walls between the two cells, undergo plasma membrane fusion, and ultimately the nuclei fuse to generate a diploid zygote (White and Rose 2001; Ydenberg and Rose 2008).

Many proteins have been identified that are required for cell fusion (Ydenberg and Rose 2008; Merlini *et al.* 2013). Fus2p, important for cell wall degradation, was first identified by a mutation that blocks cell fusion when present in both partners (Elion *et al.* 1995). Expressed in response to mating pheromone, Fus2p is initially retained in the nucleus (Paterson *et al.* 2008). Upon completion of the cell cycle, Fus2p exits the nucleus in a phosphorylation-dependent manner, and localizes at the shmoo-tip cortex (Paterson *et al.* 2008; Ydenberg and Rose 2009; Kim and Rose 2012). Mating in *fus2* mutants halts at the prezygote stage, before cell wall degradation, with vesicles accumulating at the ZCF (Gammie *et al.* 1998). Accordingly, Fus2p is thought to regulate the fusion of the vesicles with the plasma membrane to release hydrolases for cell wall breakdown (Gammie *et al.* 1998; Paterson *et al.* 2008). At the shmoo-tip, Fus2p interacts with GTP-bound Cdc42p through its Dbl-homology domain; Cdc42p function and the interaction with Fus2p are required for cell wall breakdown (Barale *et al.* 2006; Ydenberg *et al.* 2012). Cdc42p is a Rho-like GTPase with roles in polarization, signaling, and secretion throughout mating and mitosis (Richman *et al.* 1999; Johnson 1999; Kozminski *et al.* 2000; Adamo *et al.* 2001).

Rvs161p, another protein required for cell fusion, is an amphiphysin that was first identified as a mutant showing reduced viability upon starvation (Crouzet *et al.* 1991). Rvs161p is not required for viability, but mutations in the protein cause actin delocalization (Sivadon *et al.* 1995), osmotic sensitivity (Crouzet *et al.* 1991), endocytosis defects (Munn *et al.* 1995), and random budding in diploid cells (Durrens *et al.* 1995). There is another amphiphysin in yeast, Rvs167p, sharing partial homology with Rvs161p but containing an additional internal glycine-, proline-, and alanine-rich sequence (GPA) followed by a C-terminal SH3 domain (Youn *et al.* 2010). Amphiphysins are members of the BAR domain family, which includes proteins that bind cellular membranes, and promote membrane curvature by participating in membrane remodeling processes (Dawson *et al.* 2006; Youn *et al.* 2010). BAR domains mediate critical links between the actin cytoskeleton and the membrane, and are thus highly conserved across species (Dawson *et al.* 2006). The crystal structures of several BAR domains reveal that they are dimers of two monomers, each of which encompasses three α helices separated by short unstructured coils (Peter *et al.* 2004; Dawson *et al.* 2006). Kinks in the helices, together with the orientation of the two monomers, are responsible for bending of the dimeric BAR protein. The central region of the banana-shaped dimers contains an overlap of three α helices from each participating monomer to establish a six-helical bundle. The dimers have positive residues on their concave face as well as in the distal loops formed between α helices 2 and 3 in each monomer, which are important for electrostatic interactions with the negatively charged inner face of the plasma membrane (Dawson *et al.* 2006).

Rvs161p has at least two distinct and dissociable cellular functions (Brizzio *et al.* 1998). During vegetative growth and mating, Rvs161p forms a heterodimer with Rvs167p, binding membrane lipids at an early step in endocytosis (Friesen *et al.* 2006; Paterson *et al.* 2008). In mating cells, Rvs161p also binds to Fus2p, and the complex localizes to the shmoo tip in an actin- and Myo2p-dependent manner (Brizzio *et al.* 1998; Sheltzer and Rose 2009). An *rvs161*Δ *fus2*Δ double mutant does not show a more severe cell fusion defect, indicating that the two genes act together in one of the pathways responsible for cell fusion (Gammie *et al.* 1998). The interaction with Rvs161p is required for Fus2p stability; however, the Rvs161p binding domain on Fus2p is not known. Alleles of *RVS161* have been identified that separately affect endocytosis and cell fusion, indicating that these functions are at least partially independent (Brizzio *et al.* 1998). The alleles that block cell fusion are defective for binding Fus2p, but, because they are still active for endocytosis, must still interact with Rvs167p (Paterson *et al.* 2008). The formation of two exclusive complexes suggests that the binding sites for Fus2p and Rvs167p on Rvs161p at least partially overlap (Paterson *et al.* 2008). Therefore, it is likely that Rvs167p and Fus2p interact with Rvs161p in similar ways. Rvs161p interacts with the N-terminal domain of Rvs167p; both are predicted to form conserved three-helix bundle BAR domains (Navarro *et al.* 1997; Peter *et al.* 2004).

Here, we report that Fus2p binds to Rvs161p via an amphiphysin-like domain. Modeling the interaction between Rvs161p and Fus2p identified key residues involved in complex formation and function. Using function specific alleles of Rvs161p, we found that Fus2p/Rvs161p and Rvs167p/Rvs161p localize to different regions in the shmoo. Details about the Fus2p-Rvs161p interaction will fill a gap in our knowledge about a fundamental step during eukaryotic cell fusion, with relevance for several organisms.

## Materials and Methods

### General yeast methods, strain and plasmid construction

Yeast media, general methods and transformations were performed as described previously (Adams *et al.* 1997), with minor modifications. Strains and plasmids used in this study are presented in Supporting Information, Table S1 and Table S2. To generate Fus2p mutants, we introduced stop codons or point mutations into pMR5469 by the *dut ung* mutagenesis protocol (Kunkel 1985). Mutations were introduced into Rvs161p by the same approach, using plasmid pMR5912 as a template. To create Rvs161-mCherry, the mCherry fragment was amplified with primers mCherry-start.*Spe*I (5′-TAT CTA GTG AGC AAG GGC GAG GAG-3′) and mCherry-stop.*Bam*HI (5′-TAT CTG GAT CCC TTG TAC AGC TCG ACC ATG CC-3′), and digested with *Spe*I and *Bam*HI. pMR5912 was also digested with these enzymes, and the PCR fragment was ligated into the *Xba*I–*Spe*I site of the linearized vector. The resulting plasmid, pMR6588, in which Rvs161p-mCherry is expressed from the native *RVS161* promoter, was confirmed by sequencing.

Deletion mutants in *FUS2* were generated by site-directed PCR mutagenesis (Phusion, Thermo Fisher Scientific), using pMR5469 (Paterson *et al.*, 2008) as a template. Deletions were confirmed by sequencing, and pheromone-induced protein expression was confirmed by TCA precipitation followed by immunodetection (Ohashi *et al.* 1982). Fus2p C-terminal mutations were generated using the pMR5482 template, in which *FUS2* with the internal GFP tag at position 104 is expressed from its own promoter. Mutations were introduced by the *dut ung* mutagenesis protocol (Kunkel 1985).

### Coimmunoprecipitation assays

Overnight cultures were grown in selective media containing galactose, and used to start 100 ml cultures, which were grown to early exponential phase (OD_600_ = 0.2), and treated with 10 μg/ml synthetic mating pheromone (Syn/Seq Facility, Department of Molecular Biology, Princeton University) for 2 hr. Cell extracts were prepared as previously described (Brizzio *et al.* 1998). Cell pellets were resuspended in 0.75 ml breaking buffer (50 mM Tris, pH 7.4, 50 mM NaCl, and 0.5% Triton X-100) supplemented with protease inhibitors (Miniprotease tablets supplemented with 1 mM PMSF), and the cells were lysed for 1 min, three times. After clearing by centrifugation for 10 min at 13,000 rpm, lysates were incubated with 30 μl anti-FLAG M2 affinity gel (Sigma-Aldrich) that had been washed once with water, and three times with breaking buffer. Reactions were brought to 1 ml with breaking buffer, supplemented with 10 μl 5M NaCl to bring the final concentration of the reaction to 150 mM NaCl, and incubated for 1 hr at 4° with rotation. Subsequently, the beads were washed five times in breaking buffer supplemented with protease inhibitors (Roche). The protein was eluted with 100 μl SDS loading buffer, and samples were analyzed via SDS-PAGE and western blotting.

### SDS-PAGE and western blotting

Samples were prepared either by TCA precipitation (Ohashi *et al.* 1982) for protein controls, or via coimmunoprecipitiation. Proteins were resolved on SDS-PAGE gels (8% gels to visualize the Fus2p constructs, and 10% gels to visualize the Rvs161p recombinants). Proteins were then transferred to a nitrocellulose membrane (100 V, 2 hr), and, after blocking for 2 hr (3% BSA milk), the blots were incubated with primary antibody [α-GFP antibodies (Roche) at 1:1000 dilution for the Fus2p constructs, and α-FLAG antibodies (Sigma-Aldrich) at 1:5000 dilution for the Rvs161p constructs] and subsequently with secondary antibodies (α-mouse, 1:2500) for 1 hr each, and visualized by standard chemiluminescence.

### Mating assays

Limited plate mating assays were performed as described previously (Gammie and Rose 2002). For testing Rvs161p mutants, the respective plasmids were transformed into MY3909, which contains an *rvs161*::*LEU2* mutation. The strains were patched on yeast extract/peptone/dextrose (YEPD), mated with exponentially grown lawns of MY4907, which has an *rvs161*Δ *fus1*Δ double mutation, and diploids were selected by replica plating onto selective medium. For Fus2p mutants, strains were patched on YEP + 2% galactose plates, and mated with an exponential lawn of strain MY1814, which has a *fus1*Δ *fus2*Δ double mutation. After replica-plating the strains with the tester *MAT***a** strain, plates were incubated for 3 hr at 30°, and diploids were selected by replica plating to minimal medium.

For quantitative mating assays, overnight yeast cultures were diluted to early log phase and grown for approximately 4 hr. Subsequently, 3–5 × 10^6^ cells of each mating type were combined and concentrated on 2.5-cm^2^ nitrocellulose filter discs (Millipore), as previously described (Grote 2008). Cells were allowed to mate for 4 hr at 30°, and then mating mixtures were resuspended in 1 ml water or YEPD. Equal volumes of mating mixtures were plated on selective media to select for diploids and calculate the mating efficiency.

### Cell imaging

For imaging of pheromone-induced cells with fluorescent proteins, early log phase cells were treated with 10 μg/ml synthetic mating pheromone (Syn/Seq Facility, Department of Molecular Biology, Princeton University) for 1.5 hr in rich media. Cells containing a galactose-inducible protein were grown to early log phase in media containing raffinose, and then induced with pheromone (10 μg/ml), and 2% galactose for 2 hr at 30°. Pheromone-induced cells were then fixed with 2% formaldehyde for 10 min at 30°, washed twice with 1X PBS, and imaged. Mating mixtures to be imaged were prepared as above via filter mating. They were then resuspended in 1 ml of TAF buffer and imaged.

The Applied Precision Deltavision Microscopy System (Issaquah, WA) using a Nikon TE200 inverted microscope, a Photometrics Coolsnap HQ CCD camera (Tucson, AZ), and a 100X objective were used for imaging. All images were deconvolved to remove out-of-focus fluorescence. For publication purposes, the contrast and brightness were enhanced using Adobe Photoshop.

### Data availability

All strains and plasmids are available upon request. Strains and plasmids used in this study are presented in Supporting Information, Table S1 and Table S2.

## Results

### Residues of Fus2p from 415 to 626 are required for binding to Rvs161p

**Figure 1:**
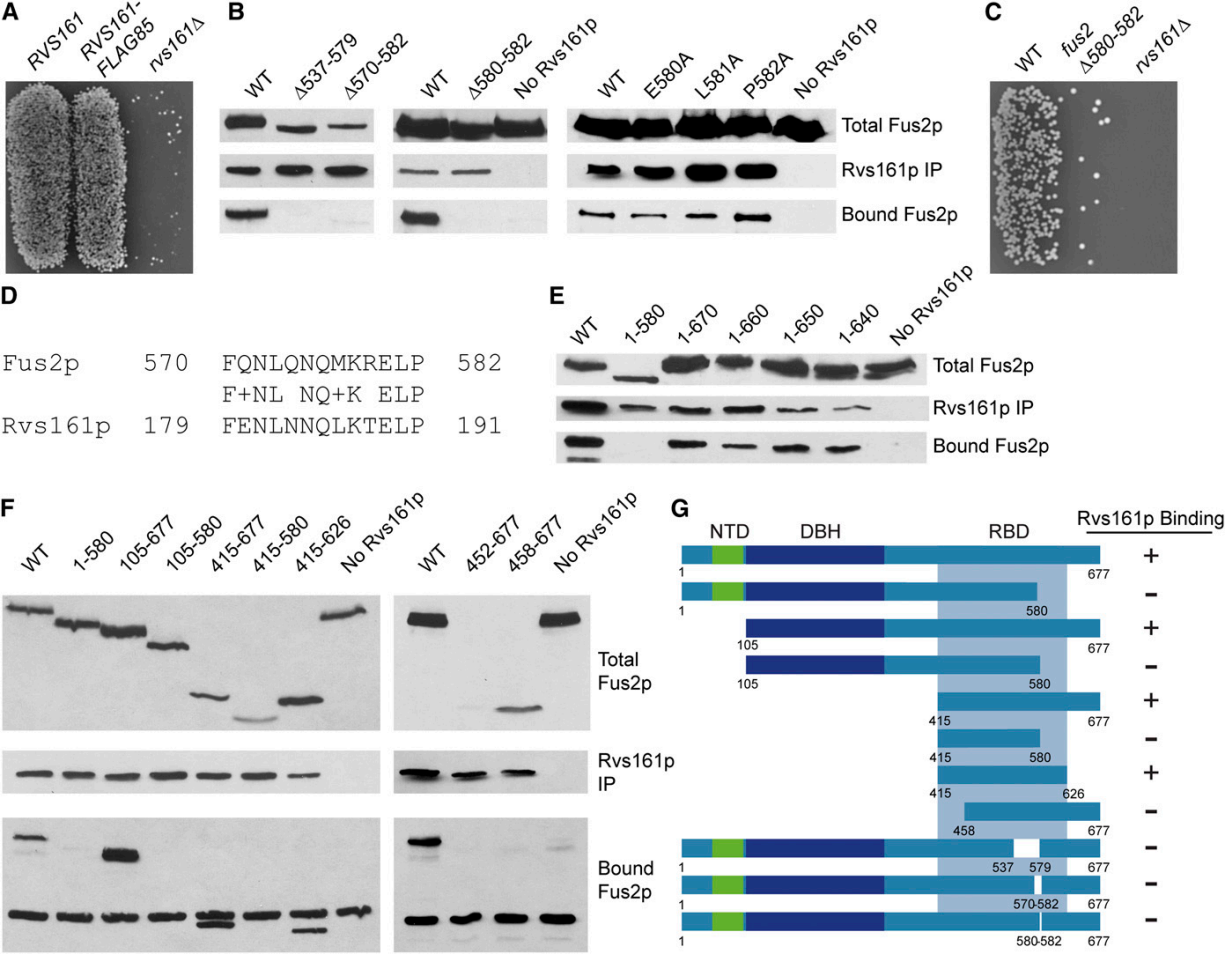
Fus2p contains an internal, amphiphysin-like Rvs161p-binding domain. (A) Rvs161p-Flag_85_ is functional. Rvs161p was internally flag tagged on either a plasmid or at its genomic locus. *rvs161∆* (MY3909) cells were transformed with plasmids containing wild-type *RVS161* (pMR3234), *RVS161-Flag_85_* (pMR5912), or an empty vector (pRS416, Sikorski and Hieter 1989), and mated to a *fus1∆ fus2∆* strain (JY429) for 3 hr at 30°. (B) Residues 537–582 are required for binding to Rvs161p. *fus2∆* cells containing genomic *RVS161-Flag_85_* (MY10904) were transformed with plasmids containing either deletions or alanine mutations introduced into pMR5469. Rvs161p was pulled down using anti-FLAG agarose beads, and bound Fus2p was assessed via western blot using anti-GFP antibodies. (C) Residues 580–582 are important during fusion. The same strains as in (B) were mated to a *fus1∆ fus2∆* strain (JY429) for 3 hr at 30°. (D) Fus2p shares homology with Rvs161p between residues 570 and 582. (E) C-terminal boundary of Rvs161p-binding domain is between residues 580 and 640. Plasmids containing C-terminal truncations made in pMR5469 were transformed into MY10904, and binding to Rvs161p was assessed as before. (F) The minimal binding domain for Rvs161p is between residues 415 and 626 in Fus2p. Coimmunoprecipitations were performed as before. (G) Map of all Fus2p fragments tested summarizing the results of the binding experiments.

To examine the structural basis of the Fus2p–Rvs161p interaction, we performed coimmunoprecipitation of the two proteins in pheromone-induced cells. For these experiments, we used tagged versions of both proteins. Fus2p, expressed from the *GAL1* promoter, was tagged with an internal GFP tag inserted in-frame after amino acid 104. The tagged protein has been shown to function like wild-type Fus2p in all assays tested (Paterson *et al.* 2008; Ydenberg and Rose 2009). Rvs161p was tagged with an internal FLAG tag, inserted after amino acid 85, in a predicted loop region that separates the first two α-helices. The tagged protein was functional in mating cells, as shown by limited plate mating (Figure 1A). As previously observed, wild-type Fus2p and Rvs161p show an interaction by coimmunoprecipitation (Figure 1, B, E, F, and G).

To determine the region of Fus2p necessary for the Rvs161p interaction, we initially generated an internal Fus2p deletion that removed 41 amino acids in the C-terminus of the protein (residues 538–579). This region of Fus2p was predicted to contain coiled-coil structures, similar to amphiphysins (Paterson *et al.* 2008). It also shares primary structure similarity with the C-terminus of Rvs161p, which contains cell fusion specific alleles (Brizzio *et al.* 1998). Within this region, we also deleted 13 residues that harbor a high degree of homology to Rvs161p (residues 570–582, Figure 1D) (Paterson *et al.* 2008). Both protein constructs lost the ability to bind to Rvs161p (Figure 1B). Within this 13-bp region were three residues, ^580^ELP^582^, that are identical in Rvs161p. We found that deletion of just these three residues (∆580–582) resulted in loss of binding to Fus2p; however, when each residue was individually mutated to alanine, binding was unaffected (Figure 1B). Consistent with the Rvs161p binding defect of Fus2p^∆580–582^, this strain showed significant mating defects (Figure 1C). We conclude that protein sequences within the C-terminal region of Fus2p, including at least residues 579–582, are required for binding to Rvs161p.

To further define the Rvs161p interaction region on Fus2p, we made additional truncations from both the N-terminal and C-terminal ends. Among the C-terminal truncations, Rvs161p was able to interact with small truncations of up to 37 amino acids, including Fus2p^1–670^, Fus2p^1–660^, Fus2p^1–650^, and Fus2p^1–640^ (Figure 1E). Interaction with Rvs161p was lost when we truncated the protein to residue 580 (Figure 1, E and F). These experiments reveal that the C-terminal border of the minimal binding domain is at, or upstream of, 640.

Among the N-terminal truncations, both Fus2p^105–677^ and Fus2p^415–677^ were able to bind Rvs161p. However, a deletion that began downstream, Fus2p^458–677^, did not interact with Rvs161p. A slightly larger fusion, Fus2p^452–677^, was too unstable to assay. The most extensive N-terminal truncation to still bind Rvs161p was Fus2p^415–677^, which places the left border of the minimal binding site at, or upstream of, residue 415 (Figure 1F). The results of all coimmunoprecipitations performed on truncations of Fus2p are summarized in Figure 1G.

**Figure 2:**
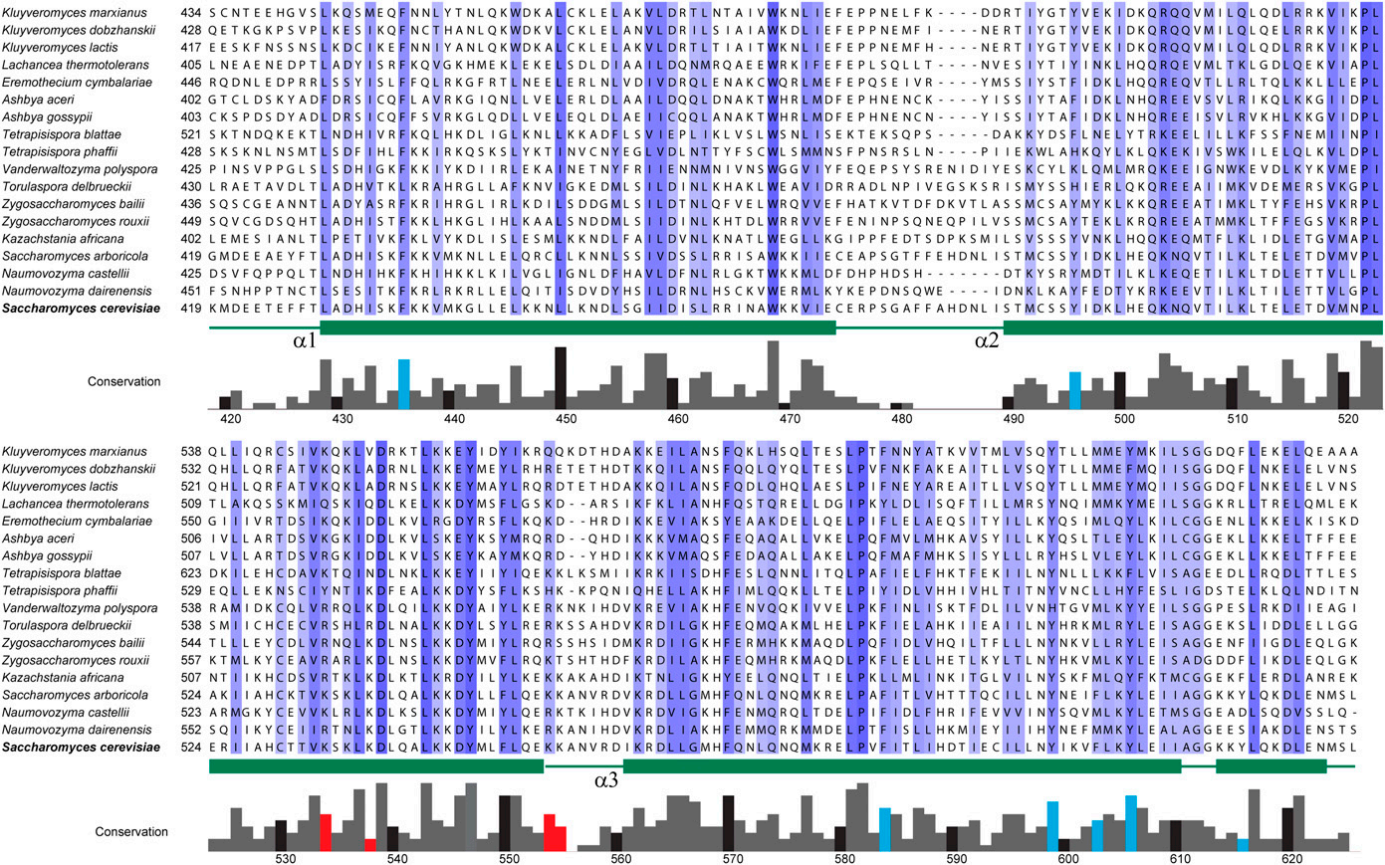
Conservation of the Rvs161p-binding domain among fungi. Residues 419–626 from *Saccharomyces cerevisiae* Fus2p were compared against homologs from other fungal species. Blue highlighting indicates the amount of conservation, with darker shaded residues being more conserved. The conservation of all amino acids is depicted using the gray bars below the residues. Black bars denote every 10 amino acids. Turquoise bars indicate the aromatic residues mutated in Figure 5; red bars indicate the lysine residues mutated in Figure 6. Predicted alpha helices in this region are mapped below the residues in green.

Comparisons of the C-terminal region of Fus2p with related fungi showed extensive conservation through residue 621 (LQKDL, Figure 2). Accordingly, to determine if the region of Fus2p between amino acids 415 and 626 might be sufficient for binding to Rvs161p, we created a protein construct with just these amino acids fused to GFP. We found that this construct bound Rvs161p similar to the wild type protein (Figure 1F). We conclude that the Fus2p^415-626^ is sufficient for binding Rvs161p.

### Modeling the Fus2p/Rvs161p interaction region as an amphiphysin-like domain

**Figure 3:**
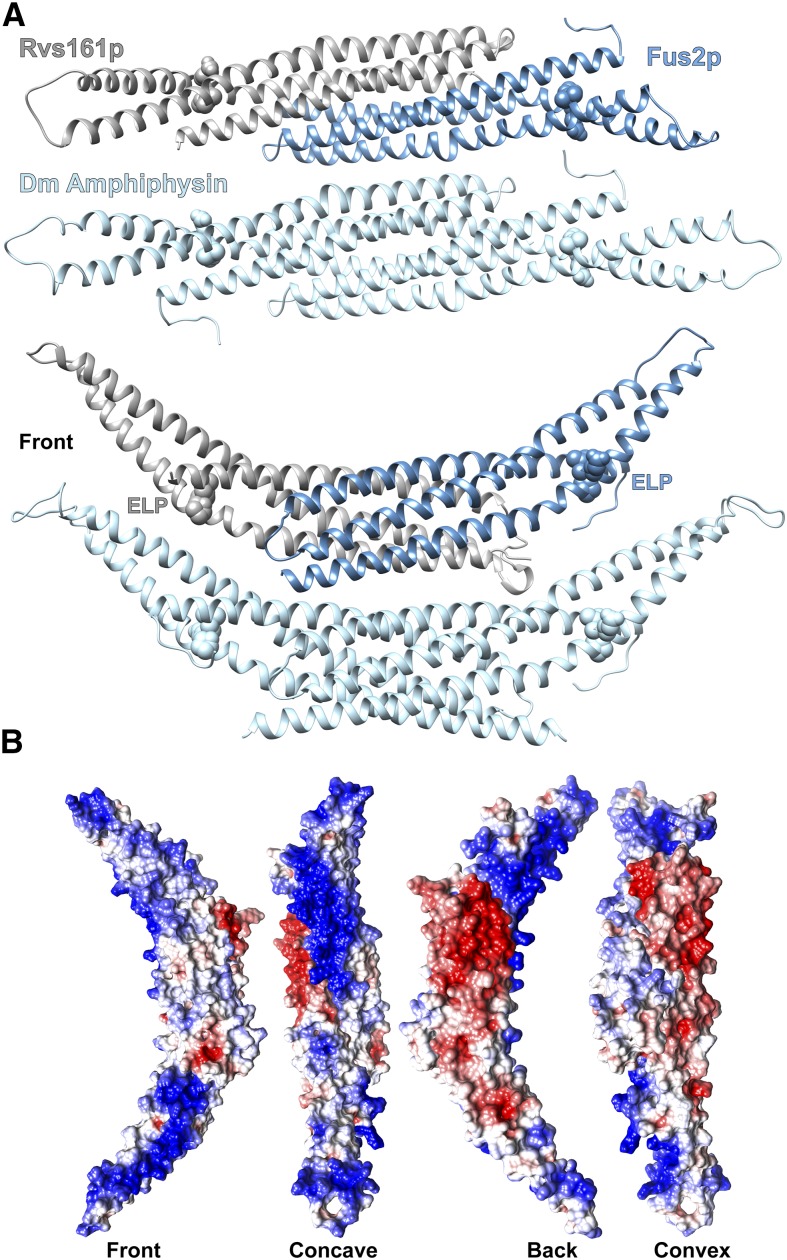
Modeling of the Fus2p-Rvs161p interaction predicts a banana-shaped heterodimer. (A) Fus2p and Rvs161p are predicted to form a heterodimer similar to the *Drosophila* (Dm) amphiphysin homodimer. The structure of Fus2p residues 415–626 (shown in blue), and Rvs161p residues 28–233 (shown in gray) were individually modeled using the PHYRE2 program (Kelley and Sternberg 2009). The two predicted structures were then modeled on the Dm amphiphysin homodimer (shown in light blue) using MatchMaker in the Chimera modeling program (Pettersen *et al.* 2004; Meng *et al.* 2006). (B) Electrostatic mapping of the Fus2p-Rvs161p heterodimer reveals surfaces with high positive charges on the front and concave faces. Basic residues are shown in blue; acidic residues are shown in red.

The three-dimensional structure of the Fus2p^415–626^ fragment was predicted using the PHYRE2 (**P**rotein **H**omology/analog**Y R**ecognition **E**ngine V 2.0) web server, which performs a structure-based sequence alignment to identify proteins of known structure that are homologous to a protein with a given sequence (Kelley and Sternberg 2009). The PHYRE server uses proteins from the Structural Classification of Proteins (SCOP) database that is augmented with more recent depositions in the Protein Data Bank (PDB) (Murzin *et al.* 1995; Berman 2000). PHYRE can reveal significant structural homology between protein pairs that harbor limited primary sequence homology, sometimes as little as 15%–25% (Kelley and Sternberg 2009). For Fus2p^415–626^, modeling provided a structure homologous with the *Drosophila* amphiphysin BAR domain, with 99.84% confidence for the three-dimensional superimposition. A different model predicted by a similar program, I-Tasser, was virtually identical in structure, differing by a root mean square deviation (RMSD) of 1.052 Ǻ (Zhang 2008; Roy *et al.* 2010; Yang *et al.* 2014). The predictions indicate that this fragment is organized into three α-helices that are joined by short loops, adopting an overall structure that is reminiscent of the α-helical structures described in amphiphysins (Figure 3A). The extents of the three predicted α-helices closely match the highest regions of conservation in the Fus2p C-terminal region (Figure 2). The same prediction algorithm was used to model Rvs161p, and residues 28–233 of the protein were modeled on the BAR domain from human Bin1/Amphiphysin II (Casal *et al.* 2006) with 100% confidence (Figure 3A).

To model the interaction between Fus2p^415–626^ and Rvs161p, we used the *Drosophila* (Dm) amphiphysin dimer, for which the N-terminal residues 1–245 have been crystallized and the structure determined at 2.6 Å resolution (Peter *et al.* 2004). The PHYRE output structures for Fus2p^415–626^ and Rvs161p were modeled on the two monomers that form the Dm amphiphysin dimer by using the homology-modeling algorithm MatchMaker in the Chimera modeling program (Pettersen *et al.* 2004; Meng *et al.* 2006). The predicted structure formed between Fus2p^415–626^ and Rvs161p creates a banana-shaped heterodimer, strikingly similar to the amphiphysin dimers that were previously characterized in other organisms (Figure 3A). The residues in the predicted Fus2p and Rvs161p chains differ from those in Dm amphiphysin by 0.127 and 0.941 Ǻ RMSD, respectively.

Combining this model with the previous binding data allows us to map the residues known to be important for binding. The Rvs161 ^189^ELP^191^, and the Fus2p ^580^ELP^582^ sequences that are important for binding and mating are highlighted on the heterodimer, as are the conserved ELP residues in the Dm amphiphysin monomers (Figure 3A). In this model, the tripeptides reside at the kink in helix 3, formed by the conserved proline. The bend in the alpha helix causes the characteristic banana shape of the heterodimer, and is therefore unsurprisingly required for binding and function.

We next created an electrostatic map of the predicted dimer using the Chimera modeling program (Pettersen *et al.* 2004; Meng *et al.* 2006), where negative residues are highlighted in red and positive residues are highlighted in blue (Figure 3B). Interestingly, there are two broad regions of the heterodimer that are predicted to be strongly positively charged, the concave inner surface and the lateral outer surface largely comprised of Fus2p. As positively charged residues play roles in binding negatively charged phospholipids, the predicted electrostatic map suggests that Fus2p/Rvs161p may interact with membranes along both its concave and lateral surfaces. This may allow Fus2p to bind to highly curved membranes along the concave surface as well as more planar membranes along the front surface.

### Residues in Rvs161p required for binding to Fus2p

**Figure 4:**
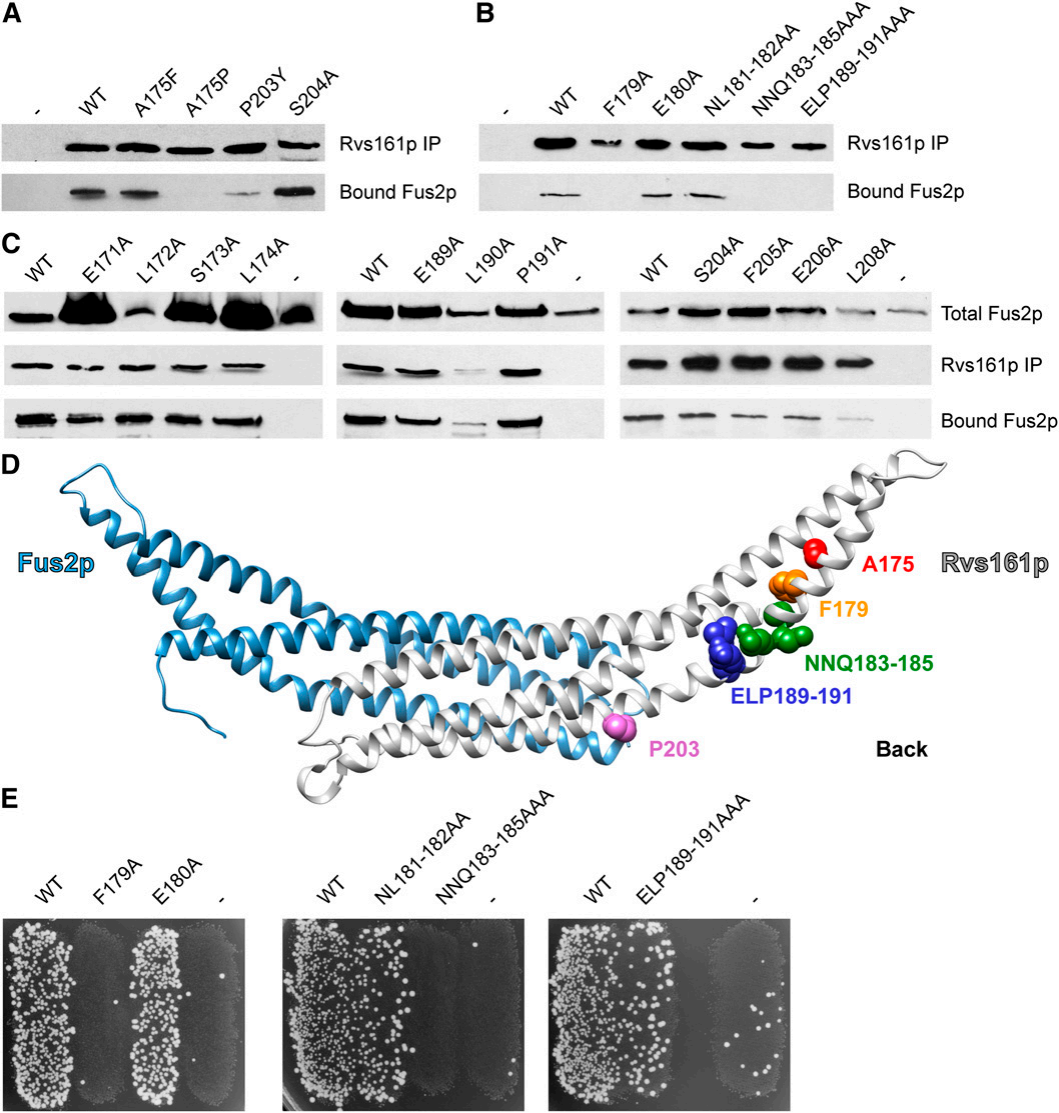
Rvs161p contains residues important for both binding to Fus2p and cell fusion. (A) Mutations of conserved residues in Rvs161p cause binding defects. A strain containing a double deletion of *FUS2* and *RVS161* (MY10463) was transformed with both a plasmid containing WT GFP-tagged *FUS2* (pMR7042) as well as alanine mutations made in *RVS161* from pMR5912. Negative controls comprised WT GFP-tagged *FUS2* and an empty vector. Binding of Fus2p to Rvs161p was assessed as before by coimmunoprecipitation. (B, C) Alanine-scanning mutagenesis revealed other residues important for binding. Alanine mutations in *RVS161* were made in pMR5912, and the mutant plasmids were transformed into MY10463 along with pMR7042. Binding was assessed as before. (D) Residues in Rvs161p at which corresponding mutations cause strongly reduced Fus2p binding. (E) Rvs161p residues important for Fus2p-binding are also important for mating. The same strains as in B were mated to a *fus1∆ fus2∆* (JY429) for 3 hr at 30°.

Previously, a genetic screen identified two amino acids in Rvs161p, A175 and P203, that are important for cell fusion (Brizzio *et al.* 1998). These residues flank the region of Rvs161p that is homologous to Fus2p (Figure 1D). The A175P and P203Q mutations disrupt binding between untagged Rvs161p and wild-type Fus2p (Brizzio *et al.* 1998). To further explore the role of this region of the protein, additional alleles in and around the conserved region were introduced into Rvs161p-Flag_85_. As found previously, A175P abolished binding, whereas A175F did not (Figure 4A). Based on the structural modeling, A175 is predicted to reside in helix 3, distal to the kink in the three-helix bundle (Figure 3C). Due to the location of this residue, it is likely that the introduction of proline at that site causes a significant change in conformation that interferes with binding. Like P203Q, the P203Y mutation, greatly decreased binding (Figure 4A). P203 is predicted to reside at the binding interface of Rvs161p and Fus2p (Figure 4C), further explaining the decrease in Fus2p binding when mutated.

Performing an alanine scan mutagenesis in Rvs161p in the region surrounding the Fus2p homology, we identified several amino acids that are important for binding Fus2p. Mutation of the highly conserved phenylalanine to alanine (F179A) abolished binding to Fus2p (Figure 4B), which is correlated with a strong mating defect. F179 resides close to A175 (Figure 4D), suggesting that this mutation may also cause a change in the conformation of Rvs161p rather than directly interfering with the interaction. Mutation of the less well-conserved neighboring residue to alanine (E180A) had no effect on binding or mating (Figure 4, B and E). Mutation of residues NL181–182 both to alanine had no effect on binding. In contrast, the NNQ183–185AAA mutation, affecting the conserved glutamine, and the ELP189–191AAA mutation, which affects the highly conserved ELP residues, abolished binding to Fus2p (Figure 4B), and resulted in strong mating defects (Figure 4E). Mutation of the NNQ tripeptide (Figure 4C) likely affects the structural integrity of Rvs161p. As for Fus2p, single mutations in ELP did not affect binding (Figure 4C). No other single site mutations (affecting residues 171–174 and 204–208) affected binding (Figure 4C).

### Conserved aromatic residues in Fus2p are important for binding to Rvs161p

Aromatic residues have been shown to play important roles in the interactions between alpha helices. These interactions are known to ensure intramolecular stability and intermolecular interactions as well as structural stability of the helices (Anderson *et al.* 1993; Butterfield *et al.* 2002). Aromatic amino acids participate in interactions that contribute to the stability of the native protein fold, and aromatic–aromatic interactions provide a mechanism to generate noncovalent interactions that ensure the stabilization of protein structures (Burley and Petsko 1985).

**Figure 5:**
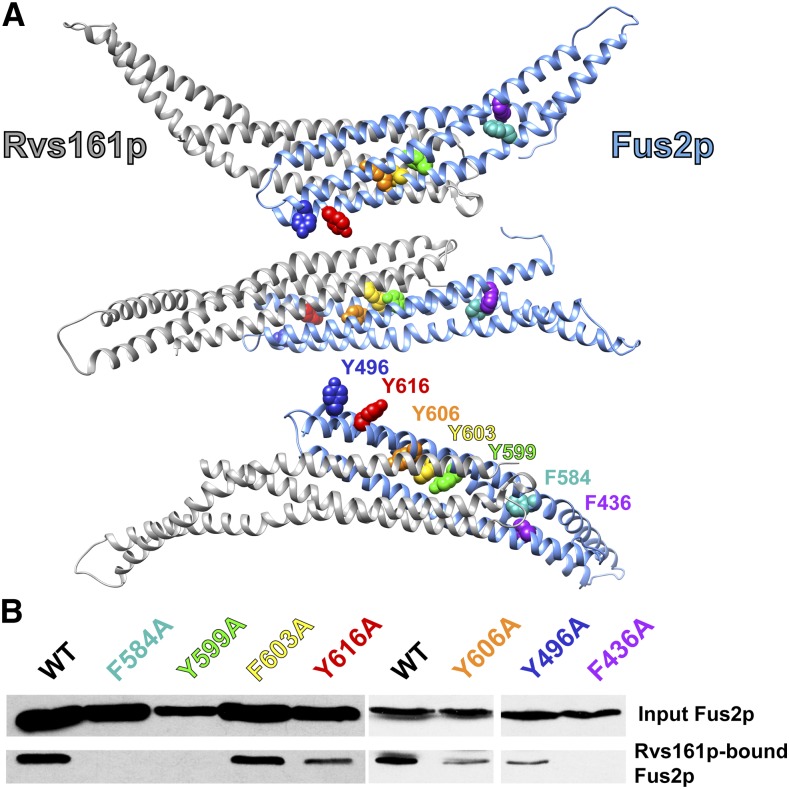
Aromatic residues in Fus2p are important for binding to Rvs161p. (A) Conserved aromatic residues are found in various orientations on the predicted Fus2p–Rvs161p heterodimer. Y496 (blue) and Y616 (red) face away from the Rvs161p interaction interface. F436 (purple) and F584 (teal) appear to face internally, but are not directly near Rvs161p. Y599 (green), Y603 (yellow), and Y606 (orange) all face into the Rvs161p–Fus2p binding interface. (B) Each aromatic residue was mutated to alanine on the plasmid containing the Fus2p 415–626 fragment (pMR6508). The resulting plasmids were transformed into MY10904 and binding assays were performed as before.

When examining the primary amino acid sequence of Fus2p^415–626^ as compared to other fungi, we found several aromatic residues that are highly conserved (Figure 2). When these residues were mapped onto Fus2p in the predicted heterodimer, several appeared to be situated in positions that could assist in binding to Rvs161p (Figure 5A). To explore the importance of these aromatic residues in binding Rvs161p, we systematically mutated individual aromatic amino acids, and tested the impact of the mutation on the interaction with Rvs161p.

Mutation of residues F436, F584 and Y599 to alanine abolished the interaction of Fus2p^415–626^ with Rvs161p (Figure 5B). Mutation of residues Y496, Y606, or Y616 to alanine decreased the interaction to various extents. However, the F603A mutation had no impact on binding (Figure 5B). These data are consistent with the conservation of the residues, as F603 and Y616 are the least conserved aromatic residues, whereas F584 and Y599 are among the most highly conserved (Figure 2). These data are also consistent with the modeling of the heterodimer. Y496 and Y616 both face away from Rvs161p, and are therefore unlikely to impact binding, while Y599 and Y606 are internal residues that reside close to the Fus2p–Rvs161p interface. F426 and F584 are distal to the Fus2p–Rvs161p binding interface, and therefore mutation of these residues likely alters the conformation of Fus2p in such a way to affect binding to Rvs161p.

### Lysine residues in the heterodimer are important for downstream function of the complex

For many proteins, membrane binding is aided by crucially positioned positively charged residues. For example, clusters of basic residues in myristoylated proteins, such as Src, MARCKS, and the HIV matrix protein, help the binding to membranes via electrostatic interactions with acidic phospholipids (McLaughlin and Aderem 1995; Ben-Tal *et al.* 1996). Conserved lysine residues in amphiphysins, located on the predicted concave side of the dimer, and on the extended loops at the ends of the dimer, were shown to be important *in vitro* for binding to liposomes and for tubulation (Peter *et al.* 2004).

**Figure 6:**
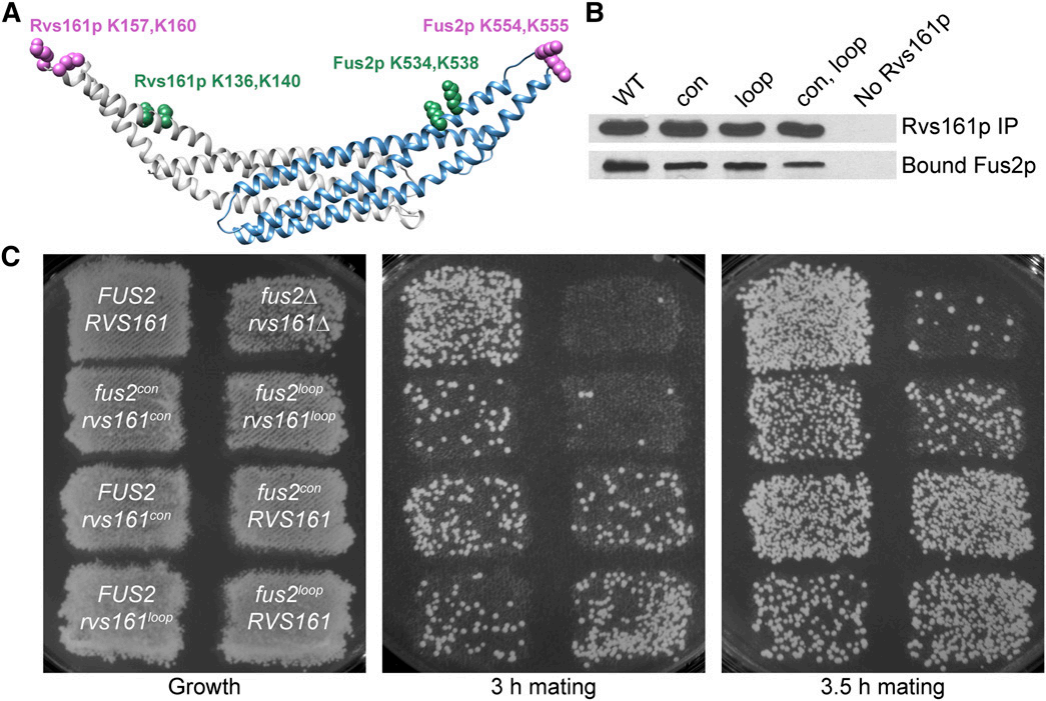
Lysine residues on the concave and loop regions of the heterodimer are important for cell fusion without affecting heterodimer formation. (A) Conserved lysine pairs highlighted on the predicted Fus2p–Rvs161p heterodimer. Rvs161p^K136, K140^ and Fus2p^K534, K538^ reside on the concave surface (“con”, highlighted in green), while Rvs161p^K157, K160^ and Fus2p^K554, K555^ reside on the loops (“loop”, highlighted in purple). (B) Mutation of the Rvs161p con and/or loop residues does not affect binding to wild-type Fus2p. Plasmids containing mutation of the con residues (pMR6543), loop residues (pMR6504), or both (pMR6512) in Rvs161p-FLAG were transformed along with wild-type Fus2p-GFP (pMR5469) into a *fus2∆ rvs161∆* strain (MY10463). Fus2p binding was assessed via coimmunoprecipitation as before. (C) Mutation of the lysine residues resulted in a cell fusion defect. Plasmids containing *RVS161* con (pMR6543) or loop (pMR6504) mutations were transformed along with plasmids containing *FUS2* con (pMR6505) or loop (pMR6507) mutations into a *fus2∆ rvs161∆* strain (MY10463). Mating efficiency was tested via mating to a *fus1∆ fus2∆* strain (JY429) for 3 or 3.5 hr at 30° to detect subtle differences in phenotypes.

Rvs161p has a highly conserved pair of lysine residues, K136 and K140 (Youn *et al.* 2010), on helix α2 located on the predicted concave surface (Figure 6A). Another pair of conserved lysine residues, K157 and K160, is located in the extended loop between helices α2 and α3. Rvs167p has similarly conserved residues on its concave surface and loop regions. Bilateral mutagenesis of the lysine residues from the concave and loop regions of Rvs161p and Rvs167p to glutamic acid has been shown to cause a severe defect in cortical localization and endocytosis (Youn *et al.* 2010). This defect is hypothesized to be due to a reduced binding of the mutant heterodimeric amphiphysin complex to membranes. Moreover, mutation of the conserved lysines on Rvs161p results in a defect in cell fusion (Youn *et al.* 2010), suggesting that membrane association may be important for Fus2p/Rvs161p function.

The structural prediction for Fus2p^415–626^ helped identify conserved lysine residues that are in similar locations to the residues in amphiphysins (Figure 6A). K534 and K538 are located on the concave surface of the heterodimer, whereas K554 and K555 are in the loop region (Figure 6A). In wild-type Fus2p, we mutated the two lysine residues from the predicted Fus2p concave surface (“con”) and, separately, the two lysine residues from its loop region (“loop”), to glutamate residues. Con and loop mutations in either Rvs161p or Fus2p caused decreased mating efficiencies compared to wild-type strains (Figure 6C). Mutation of all four loop lysines in both Fus2p and Rvs161p had a more severe phenotype than mutation of all the con lysines, suggesting that the loop residues are more important for function. When each pair of lysines on the concave surfaces were mutated in Fus2p or Rvs161p, a mating defect was still observed, although it was not as severe as in combination. The mating defects for *fus2^con^* or *rvs161^con^* were similar, suggesting that both surfaces contribute equally for fusion. However, when the equivalent experiment was performed with *fus2^loop^* and *rvs161^loop^*, mutation of Rvs161p was more deleterious than mutation of Fus2p (Figure 6C), implying that the loop residues of Rvs161p are more important for function than those of Fus2p.

To determine if the mating defect was simply due to a defect in the Fus2p–Rvs161p interaction, we performed coimmunoprecipitations using Rvs161p with the con, loop, or all four lysines (4K to 4E) mutated. All strains contained wild-type Fus2p. Although each of the mutations resulted in slightly decreased binding of Fus2p, all three constructs still bind Fus2p (Figure 6B). Therefore, we conclude that these lysine residues are important for mating efficiency without affecting heterodimer formation. These results support the conclusion of Youn *et al.* (2010) that membrane binding of Rvs161p is required for cell fusion.

### The C-terminus of Fus2p is required for localization

**Figure 7:**
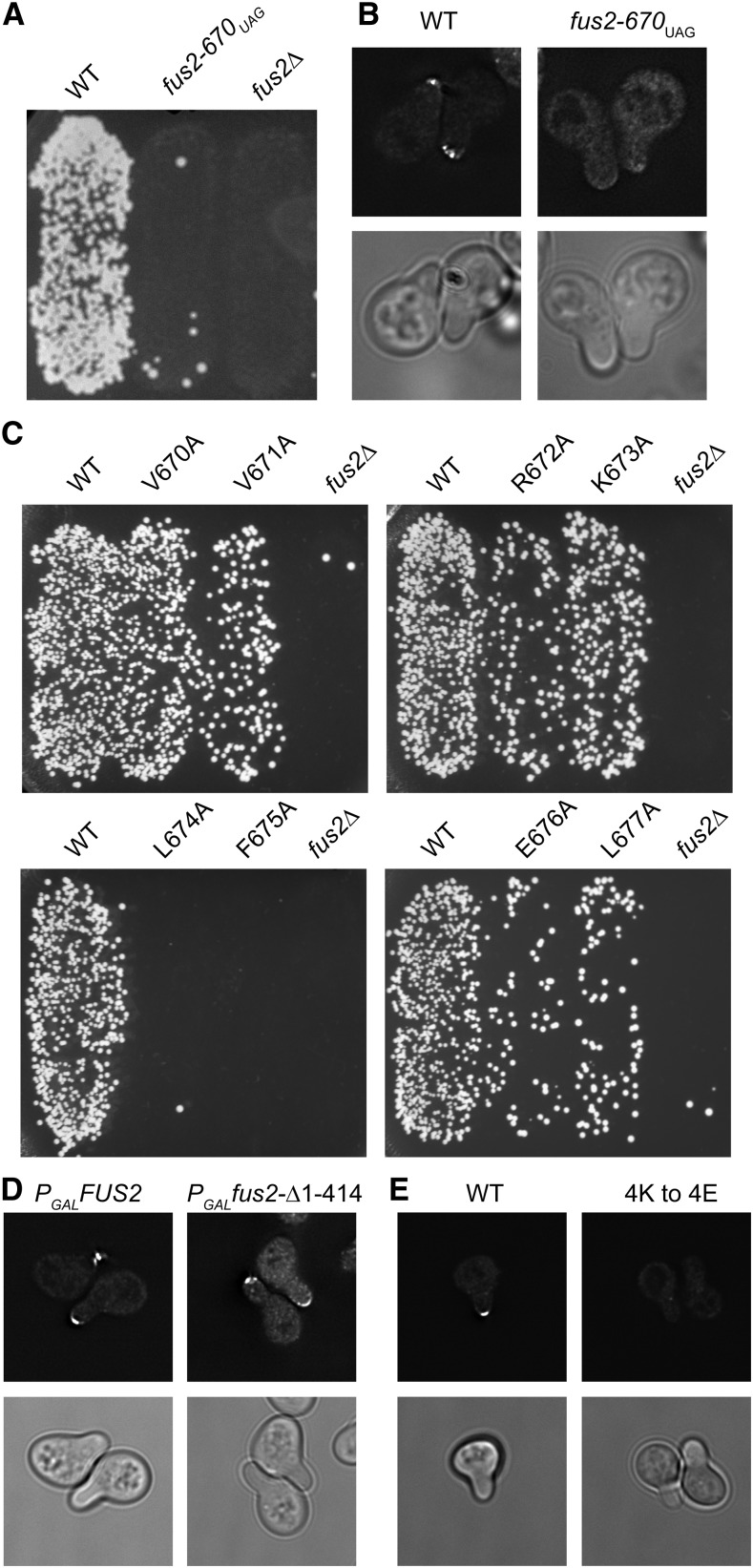
The C-terminus of Fus2p is required for cortical localization and cell fusion. (A) Mutations in the C-terminal eight amino acids of Fus2p cause defects in cell fusion. *fus2∆* cells (MY10904) were transformed with plasmids containing either WT *FUS2* (pMR5482), *fus2-670_UAG_* (pMR6775), or an empty vector (pRS416), and mated to a *fus1*∆ *fus2*∆ strain (JY429) for 3 hr at 30°. (B) Fus2p^1-670^ is defective for cortical localization. The same strains as in A were imaged after incubation with pheromone for 1.5 hr. *n* ≥ 200 shmoos imaged in three independent experiments. (C) Individual point mutations show decreased mating efficiency. Plasmids containing individual alanine mutations for the last eight amino acids were transformed into a *fus2*∆ strain (MY10904). Mating efficiency was assessed after mating to a *fus1*∆ *fus2*∆ strain (JY429) for 3 hr at 30°. (D) The C-terminus of Fus2p is sufficient for cortical localization. A plasmid containing a *FUS2* construct in which the first 414 amino acids were deleted under the control of the *GAL1* promoter was transformed into a *fus2∆* strain (MY9181). Cells were imaged after incubation with pheromone and galactose for 2 hr. *n* ≥ 250 shmoos were imaged in three independent experiments. (E) Rvs161p is required for cortical localization of Fus2p. A *fus2∆ rvs161∆* strain (MY10463) was transformed with a wild-type *FUS2-GFP* plasmid (pMR7042) as well as a plasmid containing either wild-type *RVS161* (pMR5912) or *RVS161^4K→4E^* (pMR6512). Strains were imaged after 2 hr incubation with pheromone and galactose. At least 175 shmoos were imaged in three independent experiments.

While analyzing the truncations of Fus2p used to define the binding region for Rvs161p, we observed significantly reduced mating efficiency for the strain harboring a truncation of the last eight amino acids of Fus2p, *fus2–670_UAG_* (Figure 7A). The mating defect cannot be caused by a lack of heterodimer formation as this construct binds Rvs161p as well as wild-type Fus2p (Figure 1E). Wild-type Fus2p is known to localize to the shmoo tip in pheromone-treated cells (Paterson *et al.* 2008; Ydenberg and Rose 2009). We therefore assayed the cortical localization of Fus2p^1–670^ and found that the mutant protein was diffusely localized throughout the cell (Figure 7B). These data indicate that whereas Rvs161p binding is necessary for Fus2p localization, it is not sufficient.

We individually mutated each of the eight C-terminal amino acids to alanine in otherwise wild-type Fus2p and cortical localization and mating efficiency were assessed. The point mutants showed varying mating phenotypes. Mutation of V670 and K673 did not cause a distinguishable defect. Mutation of V671, R672, E676, or L677 all caused intermediate phenotypes. Finally, mutation of L674 or F675 caused a severe mating phenotype, comparable to the C-terminal truncation (Figure 7C). The mating efficiency of each mutant was also measured by quantitative methods and compared to the defect in localization (Table 1). The two defects are strongly correlated, and all mutations that severely affected localization also resulted in a mating phenotype (Table 1). However, some mutations caused mating defects that are more severe than expected based on localization (*e.g.*, compare V671A to L677A). This observation may be understood by postulating that the mutations may cause additional cell fusion defects beyond localization.

**Table 1:** Summary of mating and localization phenotypes associated with Fus2p C-terminal mutations

*FUS2*	Mating (%)	Localization (%)
WT	> 99	> 98
∆670–677	8 ± 2	8 ± 2
V670A	52 ± 5	76 ± 3
V671A	19 ± 1	66 ± 2
R672A	6 ± 2	27 ± 6
K673A	58 ± 4	44 ± 6
L674A	< 0.1	4 ± 3
F675A	< 0.1	< 0.1
E676A	48 ± 4	55 ± 6
L677A	31 ± 34	66 ± 3

We next wanted to determine if the C-terminus of Fus2p is sufficient for localization. We began by assessing the localization of Fus2p^415–677^, which binds Rvs161p (Figure 1F), and contains the C-terminus required for localization. Fus2p^415–677^ was localized to the cortex in 70 ± 2% of cells examined (Figure 7D), showing that the NTD and DBH domains are not required for cortical localization. To determine if the Rvs161p binding domain is necessary for cortical localization, we created a construct in which residues 626–677 of Fus2p tagged to GFP was expressed from the *GAL1* promoter. However, this construct was not stable in pheromone-induced cells, and did not localize. Because Rvs161p binding is required for Fus2p stability (Brizzio *et al.* 1998), to assess the role of the C-terminus, we used the Rvs161p^4K→4E^ mutant, which blocks membrane binding, but not Fus2p binding [Figure 6 (Youn *et al.* 2010)]. The effect of the Rvs161p^4K→4E^ mutation will therefore indicate whether Rvs161p plays a role in the cortical localization of Fus2p, as well as stabilizing Fus2p, or is required only for Fus2p stability. We found that wild-type Fus2p was mislocalized in the Rvs161p^4K→4E^ mutant background, with 14 ± 3% of cells having localized Fus2p (Figure 7E). This is consistent with the observed mating defect (Figure 6C). Given that membrane-binding of Rvs161p is also required for cortical localization of Fus2p, we conclude that the C-terminus of Fus2p is necessary but not sufficient for cortical localization.

### Rvs161p is localized in two pools in pheromone-induced cells

It was shown previously that both Fus2p and Rvs161p localize to the shmoo tip and interact in pheromone-treated cells, and that this interaction is required for efficient cell fusion (Brizzio *et al.* 1998; Gammie *et al.* 1998; Paterson *et al.* 2008). However, Rvs161p also functions in a complex with Rvs167p during endocytosis, which also occurs in pheromone-induced cells (Friesen *et al.* 2006). Distinct patterns of localization to the shmoo projection have been described previously (Narayanaswamy *et al.* 2009). Proteins involved in exocytosis are localized to the shmoo-tip, whereas proteins involved in endocytosis are localized more diffusely over the entire shmoo projection. Given that there are mutant alleles that separate the two functions of Rvs161p (Brizzio *et al.* 1998), we wanted to examine the effects of these alleles on the localization of Rvs161p and Fus2p.

**Figure 8:**
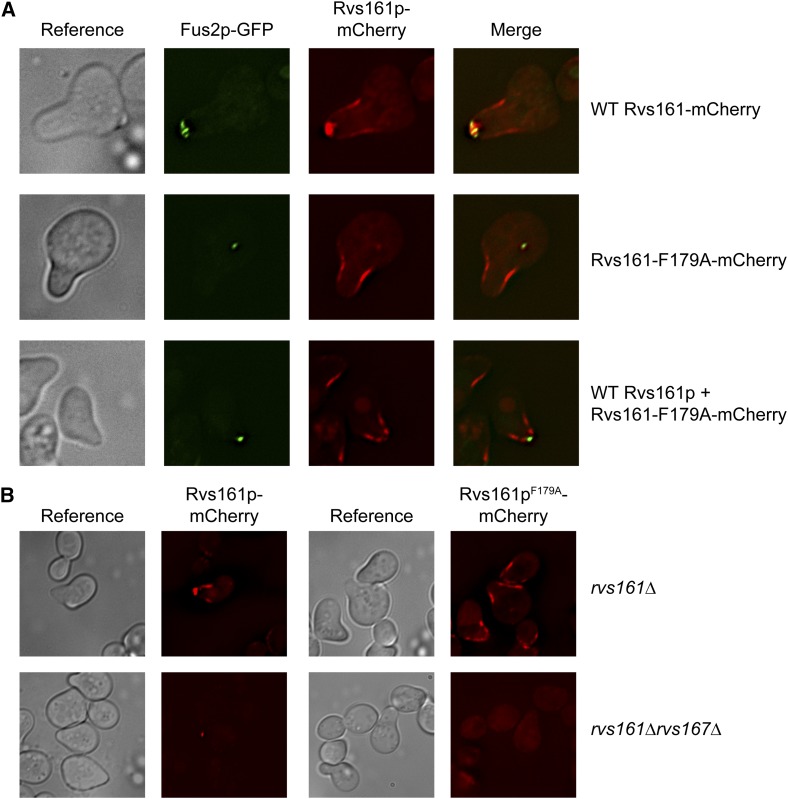
Rvs161p is localized in two pools in pheromone-induced cells. (A) Rvs161p^F179A^ causes loss of shmoo tip localization. *rvs161∆ fus2*∆ (MY10463, top two panels) or *RVS161 fus2*∆ (MY9181, bottom panel) strains were transformed with plasmids containing wild-type Fus2p-GFP (pMR7042) along with either wild-type Rvs161p-mCherry (pMR6588), or Rvs161p^F179A^-mCherry (pMR7063). Cells were imaged after incubation with pheromone for 1.5 hr. (B) The pool of Rvs161p localized at the shmoo neck is lost in *rvs167∆* cells. *rvs161∆* (MY3909) or *rvs161∆ rvs167∆* (MY4545) strains were transformed with either wild-type Rvs161p-mCherry (pMR6588), or Rvs161p^F179A^-mCherry (pMR7063) and imaged after 1.5 hr incubation with pheromone.

To examine the localization of both Rvs161p and Fus2p, we used plasmids containing Fus2p tagged with GFP at position 104 (Paterson *et al.* 2008), and created a plasmid with Rvs161p tagged internally with mCherry at position 85. Unless stated otherwise, the genomic copy of *RVS161* or *FUS2* was deleted in these strains so that the only protein observed was fluorescently tagged. As previously observed, Fus2p and Rvs161p were colocalized at the shmoo tip (Figure 8A, top panel). However, there is also a fainter pattern of localization of Rvs161p along the shmoo neck. Given that Fus2p is localized exclusively at the shmoo tip, we presume that localization along the neck of the shmoo is associated with endocytosis. To determine the effect of a lack of Rvs161p-Fus2p interaction on the localization of both proteins, we introduced the F179A mutation into Rvs161p-mCherry. This allele of Rvs161p is defective for Fus2p binding and mating (Figure 4, B and E). We found that Fus2p was not cortically localized in this strain, consistent with the requirement for Rvs161p interaction. The shmoo-tip localization of Rvs161p^F179A^ was also lost; however, the shmoo neck localization remained intact (Figure 8A, middle panel), consistent with the fact that this allele does not affect the endocytotic function of Rvs161p (Brizzio *et al.* 1998). We observed the same localization for Rvs161p^F179A^-mCherry when a wild-type, untagged copy of Rvs161p was present. In this strain, Fus2p was able to localize, by virtue of its binding to the wild-type Rvs161p; however, Rvs161p^F179A^ was still restricted to the shmoo neck (Figure 8A, bottom panel). We conclude that there are two localization pools of Rvs161p: one at the shmoo tip responsible for binding to Fus2p and cell fusion, and the other at the shmoo neck involved in endocytosis.

Because it is known that binding to Rvs167p is required for the function of Rvs161p in endocytosis (Friesen *et al.* 2006), we tested the localization of both wild-type Rvs161p and Rvs161p^F179A^ in an *rvs167*∆ background. We found that wild-type Rvs161p localization was restricted to the shmoo tip in the *rvs167*∆ mutant. When the *rvs167*∆ was combined with Rvs161p^F179A^, the mutant Rvs161p was localized diffusely throughout the strain, consistent with the loss of both binding partners (Figure 8B). We conclude that the localization of Rvs161p to the shmoo neck is dependent on Rvs167p, further supporting that this pool of Rvs161p is involved in endocytosis.

## Discussion

### Rvs161p and Fus2p form a heterodimeric amphiphysin-like complex

Amphiphysins, defined by a shared BAR (*B*in1 *A*mphiphysin *R*vs) domain, are highly conserved throughout evolution. Amphiphysins have been implicated in a variety of cellular processes including, but not limited to, endocytosis, regulation of the actin cytoskeleton, tissue differentiation, transcriptional repression, and cell–cell fusion (Ren *et al.* 2006). Despite the wide array of functions of BAR domains, they share a common mode of action in membrane remodeling. X-ray crystallography has shown that amphiphysins form an alpha-helical banana-shaped dimer, which is able to bind to curved membranes (Dawson *et al.* 2006). The majority of amphiphysins form homodimers; however, heterodimers are also observed, as in the case of the Rvs161p–Rvs167p complex in *S. cerevisiae* (Youn *et al.* 2010). Rvs161p also forms a heterodimer with Fus2p in mating cells, and these two complexes have very different functions (Brizzio *et al.* 1998). The preference for formation of homodimers *vs.* heterodimers may provide insight into the diversity of functions performed by an amphiphysin protein.

BAR-domain-containing proteins have been shown to sense curvature, and/or create curvature in lipid bilayers. Amphiphysin dimers can bind to the negatively charged membrane via clustering of positive residues along one face of the structure (Dawson *et al.* 2006). The Rvs161p–Rvs167p complex has been shown to oligomerize during endocytosis, and form helical structures at the neck of the forming endocytic vesicle (Engqvist-Goldstein and Drubin 2003; Youn *et al.* 2010). Constriction of the invaginating endocytic tubule by Rvs161p–Rvs167p is thought to help drive vesicle scission (Kishimoto *et al.* 2011). During mating, the Rvs161p–Fus2p complex localizes to the shmoo tip, where it acts with GTP-bound Cdc42p to promote cell fusion (Brizzio *et al.* 1998; Paterson *et al.* 2008; Ydenberg *et al.* 2012). This complex is thought to aid the fusion of glucanase-bearing vesicles with the plasma membrane. Prior to cell fusion, Rvs161p-Fus2p may localize to the essentially planar plasma membrane at the shmoo tip, as opposed to the highly curved endocytic tubules. However, after cell fusion, Fus2p forms an expanding ring around the cell fusion pore, possibly by binding to the highly curved membrane along the edge. The dual requirements for membrane localization before and after cell fusion may account for the striking pattern of high positive charge on both the front and concave faces of the Rvs161p-Fus2p heterodimer.

### Rvs161p is required for Fus2p localization and stability

Previous work showed that mutations in conserved lysine residues on the surface of the amphiphysin dimer affect the ability of the complex to bind membranes (Youn *et al.* 2010). Equivalent lysine mutations in Rvs161p cause cell fusion defects indicating the importance for membrane binding in this pathway (Youn *et al.* 2010). Similarly, mutations of the conserved lysines in Fus2p also block cell fusion, without affecting heterodimer formation (Figure 6). Thus membrane binding of both partners in the complex is likely to be important for cell fusion.

The Rvs161p–Fus2p interaction is required for the stability of Fus2p (Brizzio *et al.* 1998; Paterson *et al.* 2008), which complicates assessment of any role for Rvs161p in retaining Fus2p at the shmoo tip. To address this question, we studied wild-type Fus2p localization in the Rvs161p^4E^ mutant background. In this strain, Rvs161p has all four surface lysines mutated to glutamic acid, which causes cell fusion defects without affecting Fus2p binding. We found that wild-type Fus2p is stable but mislocalized in this strain. We conclude that membrane interaction of Rvs161p is required for the cortical localization of Fus2p (Figure 7).

We found that the C-terminus of Fus2p is also required for localization, indicating that the Rvs161p interaction is not sufficient for localization. Therefore, stable cortical localization is dependent on multiple interactions, any one of which is not sufficient. Presumably, the Rvs161p binding domain binds directly to the plasma membrane, whereas the C-terminus of Fus2p interacts with other cortical proteins. It has been shown previously that Fus2p is retained at the shmoo tip dependent both on Fus1p and polymerized actin (Paterson *et al.* 2008; Sheltzer and Rose 2009). We hypothesize that these partially redundant pathways may be acting through the C-terminus.

Although Rvs161p localized at the shmoo tip in mating cells, it was also localized at puncta along the shmoo neck. The shmoo tip localization was Fus2p-dependent, whereas the shmoo neck localization was Rvs167p-dependent (Figure 8). Therefore, the two localization sites for Rvs161p spatially segregate its two functions. Cell fusion activity would be restricted to the shmoo tip, and the housekeeping function of endocytosis would occur along the sides of the shmoo projection. The spatial restriction of proteins required for cell fusion may help ensure that conjugation occurs only between two partners, ensuring the production of diploid zygotes.

### Potential conservation of cell fusion proteins and mechanisms

Cell fusion events are ubiquitous among eukaryotes, raising the question of whether the basic mechanism and proteins required for fusion are conserved. Post fertilization, many fusion events occur during mammalian development, with one of the best-characterized events being myoblast fusion during muscle formation. In *Drosophila*, myoblast fusion is asymmetric, with muscle founder cells fusing with fusion competent myoblasts (FCMs). The FCMs produce actin-rich, finger-like, protrusions into the founder cells, inducing inward curvature on the founder cell membrane. The protrusions are surrounded by adhesion molecules required for fusion, and closely resemble podosomes. Podosomes are actin-dependent protrusions that are associated with extracellular matrix degradation, through secretion of matrix metalloproteinases (Linder 2007). The podosome-like structure (PLS) in myoblast fusion pushes the two membranes into closer proximity, allowing for increased surface contact of the opposing membranes (Kim *et al.* 2015). It is not known whether the PLS is also required to degrade extracellular matrix separating the two cells. Regardless, the PLS resembles the shmoo tip in *S. cerevisiae* in being an actin-dependent, actin-enriched cell fusion structure. Recent work demonstrated a similar “actin fusion focus” in the fission yeast, *Schizosaccharomyces pombe*. Like the PLS, the fusion focus is asymmetric and Arp2/3-dependent (Dudin *et al.* 2015).

It is likely that curved membrane binding proteins may be required during fusion to stabilize the finger-like protrusions, and/or facilitate membrane fusion. Indeed, intracellular curvature-generating proteins, including BAR-domain-containing proteins, facilitate hemagglutinin-promoted cell–cell fusion (Richard *et al.* 2011). In mice, GRAF1, a BAR-domain-containing GTPase-activating protein, is required for myoblast fusion and overexpression of GRAF1 in cultured myoblasts induces cell fusion (Kim *et al.* 2015). A second BAR domain protein, Bin3, is also required for mouse muscle myogenesis and myotube formation (Simionescu-Bankston *et al.* 2013). Interestingly, Bin3 is an ortholog of Rvs161p, and, like Rvs161p, contains only an N-BAR domain (Ren *et al.* 2006). Moreover, myotube formation requires actin polymerization dependent on the small GTPases, Rac1, and Cdc42 (Vasyutina *et al.* 2009). Bin3 forms a complex with active Rac1 or Cdc42 (Simionescu-Bankston *et al.* 2013). Because Bin3 only contains a BAR domain, it is hypothesized to interact with other proteins that bind the GTPases, similar to the Rvs161p–Fus2p complex. Mouse Toca-1, an F-BAR protein, also affects myoblast fusion. Toca-1 interacts with Cdc42, and activates Cdc42-mediated actin nucleation through interaction with the Arp2/3 activator N-WASP (Ho *et al.* 2004; George *et al.* 2014). Thus, in both mice and yeast, BAR domain proteins are required for cell fusion.

Cdc42p is required at two stages in yeast cell fusion. First, it is required for cell polarization and formation of the shmoo tip (Barale *et al.* 2006). Later, the Rvs161p–Fus2p complex binds GTP-bound Cdc42p to promote cell fusion (Ydenberg *et al.* 2012). Proteins homologous to Cdc42p are implicated in *Drosophila*, mouse, and zebrafish myoblast fusion. In *Drosophila*, the GTPase Rac activates the Scar complex, which promotes actin polymerization via the Arp2/3 complex. Additionally, both the small GTPase Rac1 and Cdc42 have been shown to play a role in mouse myoblast fusion *in vivo* (Vasyutina *et al.* 2009).

Invadosomes are protrusive F-actin structures, similar to podosomes, that promote tumor cell invasion via degradation of the ECM. ”Linear” invadosomes form specifically upon contact with type I collagen fibrils. Interestingly, whereas Rho GTPases are involved in classical invadosome formation, only Cdc42p is required for the formation of linear invadosomes. Tuba is a BAR domain protein that acts as a linear invadosome-specific GEF for Cdc42p. Tuba contains an internal DBL homology domain, which makes it structurally the most similar to Fus2p. However, Fus2p localizes GTP-bound Cdc42p rather than activating it (Juin *et al.* 2014).

Taken together, we perceive a common conserved pathway for all of these fusion events. Actin-dependent polymerization mediated by a small GTPase (Cdc42p or Rac1) causes cells to form podosome-like protrusions. In fungi, the protrusion is required for the cells to make contact; in animal cells, the protrusions may be required to deform the membranes of the fusing cells. The tip of the protrusion is also likely to be a site where extracellular matrix is degraded. In fungi, the ECM is cell wall, whereas in animal cells it comprises collagen and other proteins degraded by podosomes. Finally, many of the membrane functions of the protrusion are mediated by amphiphysins, which serve to regulate and localize the G-proteins that mediate cell fusion.

## Supplementary Material

Supporting Information
