# Supplementary material for: An Amphiphysin-Like Domain in Fus2p Is Required for Rvs161p Interaction and Cortical Localization
Source: G3 (Bethesda). 2015 Dec 16;6(2):337–49. doi: 10.1534/g3.115.023960 (PMC4751553; doi:10.1534/g3.115.023960)
Supplement: Supporting Information [file supp_6_2_337__index.html]

An Amphiphysin-Like Domain in Fus2p Is Required for Rvs161p Interaction and Cortical Localization — Supporting Information 

# An Amphiphysin-Like Domain in Fus2p Is Required for Rvs161p Interaction and Cortical Localization

## Supporting Information for Stein, Smith, and Rose, 2016

**Files in this Data Supplement:**

- Table S1 - Yeast strains used in this study. (.pdf, 65 KB)
- Table S2 - Plasmids used in this study. (.pdf, 86 KB)
