# Supplementary material for: An Amphiphysin-Like Domain in Fus2p Is Required for Rvs161p Interaction and Cortical Localization
Source: G3 (Bethesda). 2015 Dec 16;6(2):337–49. doi: 10.1534/g3.115.023960 (PMC4751553; doi:10.1534/g3.115.023960)
Supplement: Supporting Information [file supp_g3.115.023960_TableS1.pdf]

Table S1. Yeast strains used in this study

Strain	Genotype	Source
JY429	*MAT*α *trp1-d1 ura3-52 cyh2 fus1-d1 fus2-d3*	G. Fink (Whitehead Institute, Cambridge, MA)
MY3909	*MAT***a** *rvs161*Δ*1::LEU2 ura3-52 leu2*Δ*1 his3*Δ*200 trp1*Δ*63*	(BRIZZIO *et al.* 1998)
MY4545	*MAT***a** *rvs161*Δ*1::LEU2 rvs167*Δ*1::HIS3 ura3-52 leu2 his3*Δ*200 trp1*Δ*63*	(BRIZZIO *et al.* 1998)
MY9181	*MAT***a** *fus2::HIS3 ura3*Δ*0 leu2*Δ*0 his3*Δ*1 met15*Δ*0*	(PATERSON *et al.* 2008)
MY10904	*MAT***a** *fus2::HIS3 RVS161-Flag85 ura3*Δ*0 leu2*Δ*0 his3*Δ*1 met15*Δ*0*	
MY10463	*MAT***a** *fus2::HIS3 rvs161::NatMX CDC3-chFP::KanMX ura3*Δ*0 leu2*Δ*0 his3*Δ*1 met15*Δ*0*	
