# Supplementary material for: An Amphiphysin-Like Domain in Fus2p Is Required for Rvs161p Interaction and Cortical Localization
Source: G3 (Bethesda). 2015 Dec 16;6(2):337–49. doi: 10.1534/g3.115.023960 (PMC4751553; doi:10.1534/g3.115.023960)
Supplement: Supporting Information [file supp_g3.115.023960_TableS2.pdf]

Table S2. Plasmids used in this study.

Strain	Genotype	Reference
pMR3234	*RVS161 URA3 CEN3 amp^R*	(Brizzio *et al.* 1998)
pMR5469	*pGAL1-FUS2-GFP_{104} URA3 CEN3 amp^R*	(Paterson *et al.* 2008)
pMR5482	*FUS2-GFP_{104} URA3 CEN3 amp^R*	(Paterson *et al.* 2008)
pMR5784	*pGAL1-GFP-$FUS2^{105\text{-}677}$ URA3 CEN3 amp^R*	
pMR5884	*pGAL1-GFP-$FUS2^{415\text{-}677}$ URA3 CEN3 amp^R*	
pMR5886	*pGAL1-$FUS2^{1\text{-}580}$-GFP_{104} URA3 CEN3 amp^R*	
pMR5912	*RVS161-$Flag_{85}$ URA3 CEN3 amp^R*	
pMR6493	*$FUS2^{V671A}$-GFP_{104} URA3 CEN3 amp^R*	
pMR6494	*pGAL1-$FUS2^{E580A}$-GFP_{104} URA3 CEN3 amp^R*	
pMR6495	*$RVS161^{E206A}$-$Flag_{85}$ URA3 CEN3 amp^R*	
pMR6497	*$FUS2^{R672A}$-GFP_{104} URA3 CEN3 ARS1 amp^R*	
pMR6499	*pGAL1-$FUS2^{1\text{-}650}$-GFP_{104} URA3 CEN3 amp^R*	
pMR6501	*$FUS2^{L674A}$-GFP_{104} URA3 CEN3 amp^R*	
pMR6504	*$RVS161^{K157E,\ K160E}$-$Flag_{85}$ URA3 CEN3 amp^R*	
pMR6505	*pGAL1-$FUS2^{K534E,\ K538E}$-GFP_{104} LEU2 CEN3 amp^R*	
pMR6507	*pGAL1-$FUS2^{K554E,\ K555E}$-GFP_{104} LEU2 CEN3 amp^R*	
pMR6508	*pGAL1-GFP-$FUS2^{415\text{-}626}$ URA3 CEN3 amp^R*	
pMR6511	*pGAL1-GFP-$FUS2^{105\text{-}580}$ URA3 CEN3 amp^R*	
pMR6512	*$RVS161^{K136E,\ K140E,\ K157E,\ K160E}$-$Flag_{85}$ URA3 CEN3*	

amp^{R}

pMR6514 *RVS161*^{E171A}*-Flag*$_{85}$ *URA3 CEN3 amp*^{R}

pMR6516 *RVS161*^{L172A}*-Flag*$_{85}$ *URA3 CEN3 amp*^{R}

pMR6518 *pGAL1-GFP-FUS2*$^{415\text{-}626,\ F496A}$ *URA3 CEN3 amp*^{R}

pMR6519 *RVS161*$^{N181A,\ L182A}$*-Flag*$_{85}$ *URA3 CEN3 amp*^{R}

pMR6522 *RVS161*^{L208A}*-Flag*$_{85}$ *URA3 CEN3 amp*^{R}

pMR6523 *RVS161*^{A175P}*-Flag*$_{85}$ *URA3 CEN3 amp*^{R}

pMR6524 *pGAL1-FUS2*^{L581A}*-GFP*$_{104}$ *URA3 CEN3 amp*^{R}

pMR6526 *RVS161*^{N181A}*-Flag*$_{85}$ *URA3 CEN3 amp*^{R}

pMR6528 *RVS161*^{P203Y}*-Flag*$_{85}$ *URA3 CEN3 amp*^{R}

pMR6529 *RVS161*$^{N183A,\ N184A,\ Q185A}$*-Flag85 URA3 CEN3*
amp^{R}

pMR6532 *FUS2*^{K673A}*-GFP*$_{104}$ *URA3 CEN3 ARS1 amp*^{R}

pMR6534 *FUS2*^{L677A}*-GFP*$_{104}$ *URA3 CEN3 ARS1 amp*^{R}

pMR6535 *pGAL1-FUS2*$^{\Delta 570\text{-}582}$*-GFP*$_{104}$ *URA3 CEN3 amp*^{R}

pMR6536 *pGAL1-GFP-FUS2*$^{415\text{-}626,\ Y606A}$ *URA3 CEN3 amp*^{R}

pMR6537 *pGAL1-FUS2*$^{1\text{-}670}$*-GFP*$_{104}$ *URA3 CEN3 amp*^{R}

pMR6538 *RVS161*^{E180A}*-Flag*$_{85}$ *URA3 CEN3 amp*^{R}

pMR6539 *RVS161*^{F205A}*-Flag*$_{85}$ *URA3 CEN3 amp*^{R}

pMR6540 *RVS161*^{A175F}*-Flag*$_{85}$ *URA3 CEN3 amp*^{R}

pMR6542 *RVS161*^{E171A}*-Flag*$_{85}$ *URA3 CEN3 amp*^{R}

pMR6543 *RVS161*$^{K136E,\ K140E}$*-Flag*$_{85}$ *URA3 CEN3 amp*^{R}

pMR6547 *FUS2*^{V670A}*-GFP*$_{104}$ *URA3 CEN3 ARS1 amp*^{R}

pMR6548	*FUS2*^{E676A}*-GFP*$_{104}$ *URA3 CEN3 ARS1 amp*^{R}
pMR6550	*FUS2*^{F675A}*-GFP*$_{104}$ *URA3 CEN3 ARS1 amp*^{R}
pMR6551	*RVS161*^{S204A}*-Flag*$_{85}$ *URA3 CEN3 amp*^{R}
pMR6553	*pGAL1-FUS2*$^{\Delta 537\text{-}579}$*-GFP*$_{104}$ *URA3 CEN3 amp*^{R}
pMR6554	*RVS161*^{L190A}*-Flag*$_{85}$ *URA3 CEN3 amp*^{R}
pMR6588	*RVS161-mCherry*$_{85}$ *URA3 CEN3 amp*^{R}
pMR6598	*pGAL1-FUS2*$^{1\text{-}660}$*-GFP*$_{104}$ *URA3 CEN3 amp*^{R}
pMR6599	*pGAL1-FUS2*$^{1\text{-}640}$*-GFP*$_{104}$ *URA3 CEN3 amp*^{R}
pMR6600	*RVS161*$^{E180A,\ L181A,\ P182A}$*-Flag*$_{85}$ *URA3 CEN3 amp*^{R}
pMR6775	*FUS2*$^{1\text{-}670}$*-GFP*$_{104}$ *URA3 CEN3 ARS1 amp*^{R}
pMR7042	*pGAL1-FUS2-GFP*$_{104}$ *LEU2 CEN3 amp*^{R}
pMR7048	*pGAL1-FUS2*^{P682A}*-GFP*$_{104}$ *URA3 CEN3 amp*^{R}
pMR7049	*pGAL1-GFP-FUS2*$^{415\text{-}580}$ *URA3 CEN3 amp*^{R}
pMR7050	*RVS161*^{F179A}*-Flag*$_{85}$ *URA3 CEN3 amp*^{R}
pMR7051	*RVS161*^{P191A}*-Flag*$_{85}$ *URA3 CEN3 amp*^{R}
pMR7052	*pGAL1-GFP-FUS2*$^{415\text{-}626,\ F584A}$ *URA3 CEN3 amp*^{R}
pMR7053	*pGAL1-GFP-FUS2*$^{415\text{-}626,\ Y599A}$ *URA3 CEN3 amp*^{R}
pMR7054	*pGAL1-GFP-FUS2*$^{415\text{-}626,\ F603A}$ *URA3 CEN3 amp*^{R}
pMR7055	*pGAL1-GFP-FUS2*$^{415\text{-}626,\ Y616A}$ *URA3 CEN3 amp*^{R}
pMR7056	*pGAL1-GFP-FUS2*$^{415\text{-}626,\ F436A}$ *URA3 CEN3 amp*^{R}
pMR7059	*pGAL1-FUS2*$^{\Delta 580\text{-}582}$*-GFP*$_{104}$ *URA3 CEN3 amp*^{R}
pMR7060	*pGAL1-GFP-FUS2*$^{452\text{-}677}$ *URA3 CEN3 amp*^{R}
pMR7061	*pGAL1-GFP-FUS2*$^{458\text{-}677}$ *URA3 CEN3 amp*^{R}

pMR7062	*RVS161*^{S173A}*-Flag*$_{85}$ *URA3 CEN3 amp*^{R}	
pMR7063	*RVS161*^{F179A}*-mCherry*$_{85}$ *URA3 CEN3 amp*^{R}	
pRS415	*LEU2 CEN3 amp*^{R}	(SIKORSKI and HIETER 1989)
pRS416	*URA3 CEN3 amp*^{R}	(SIKORSKI and HIETER 1989)
